# *QuickStats:* Age-Adjusted Death Rates* from Prostate Cancer,^†^ by Race/ Ethnicity — National Vital Statistics System, United States, 1999–2017

**DOI:** 10.15585/mmwr.mm6823a4

**Published:** 2019-06-14

**Figure:**
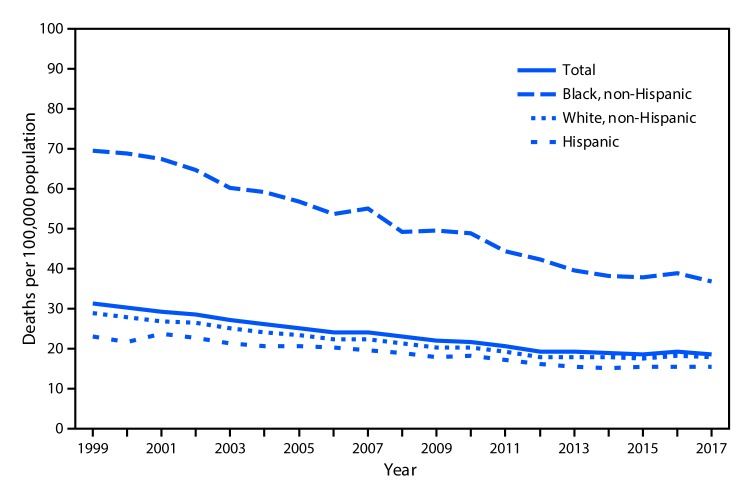
In 2017, the age-adjusted prostate cancer death rate among all males was 18.7 per 100,000, down from 31.3 in 1999. During 1999–2017, non-Hispanic black males had the highest prostate cancer death rate. In 2017, the rate for non-Hispanic black males was 36.8, compared with 17.8 for non-Hispanic white males and 15.4 for Hispanic males.

**Source:** National Vital Statistics System, Mortality, 1999–2017. https://wonder.cdc.gov/ucd-icd10.html.

